# Advances in the interaction between lumbar intervertebral disc degeneration and fat infiltration of paraspinal muscles: critical summarization, classification, and perspectives

**DOI:** 10.3389/fendo.2024.1353087

**Published:** 2024-06-24

**Authors:** Jiaqiu Jiang, Yilong Huang, Bo He

**Affiliations:** Department of Medical Imaging, The First Affiliated Hospital of Kunming Medical University, Kunming, China

**Keywords:** low back pain, intervertebral disc degeneration, paraspinal muscles, fat infiltration, interplay mechanisms

## Abstract

More than 619 million people in the world suffer from low back pain (LBP). As two potential inducers of LBP, intervertebral disc degeneration (IVDD) and fat infiltration of paraspinal muscles (PSMs) have attracted extensive attention in recent years. So far, only one review has been presented to summarize their relationship and relevant mechanisms. Nevertheless, it has several noticeable drawbacks, such as incomplete categorization and discussion, lack of practical proposals, etc. Consequently, this paper aims to systematically summarize and classify the interaction between IVDD and fat infiltration of PSMs, thus providing a one-stop search handbook for future studies. As a result, four mechanisms of IVDD leading to fat infiltration of PSMs and three mechanisms of fat infiltration in PSMs causing IVDD are thoroughly analyzed and summarized. The typical reseaches are tabulated and evaluated from four aspects, i.e., methods, conclusions, benefits, and drawbacks. We find that IVDD and fat infiltration of PSMs is a vicious cycle that can promote the occurrence and development of each other, ultimately leading to LBP and disability. Finally, eight perspectives are proposed for future in-depth research.

## 1 Introduction

Low back pain (LBP) has been identified as a major global public health problem (1) and the leading cause of the disease burden worldwide (2). According to statistics, up to 2020, approximately 619 million people around the world suffer from LBP (3). Given its prevalence and burden rise with age (4), as well as the increasing population aging and the modern lifestyle changes, LBP should be urgently addressed in the 21st century.

The pathogenesis of LBP is diverse, including intervertebral disc degeneration (IVDD), radicular pain, facet arthropathy, myofascial pain, sacroiliac joint pain, spondyloarthropathies, etc. More details are available in the review by Knezevic et al. (5). Among them, IVDD is the main pathogenic factor of LBP (6, 7). It generally exhibits disc cell apoptosis, annulus fibrosus (AF) tears, and nucleus pulposus (NP) protrusion, structural changes in the cartilaginous endplates (CEP), disc collapse, and metabolic changes (8, 9). These degenerative changes contribute to the loss of normal structure and function of the intervertebral disc (IVD), giving rise to disc-related pain and inflammation, along with decreased spinal stability (10, 11). Additionally, during disc degeneration, structure remodeling of paraspinal muscles (PSMs) usually occurs, e.g., fat infiltration, muscle asymmetry, and size reduction (12). Notably, fat infiltration of PSMs increases with the progression of IVDD and is actively associated with the severity and dysfunction of LBP (13, 14).

In fact, there is a close interaction between fat infiltration in PSMs and IVDD (12, 15). On the one hand, IVDD can induce fatty infiltration of PSMs by affecting their loading (16) and innervation (17, 18), as well as releasing inflammatory mediators (19, 20). On the other hand, fat-infiltrated PSMs can promote IVDD via increasing mechanical stress on the disc (21) and releasing inflammatory (22) and adipokine factors (23).

**Figure 1:**
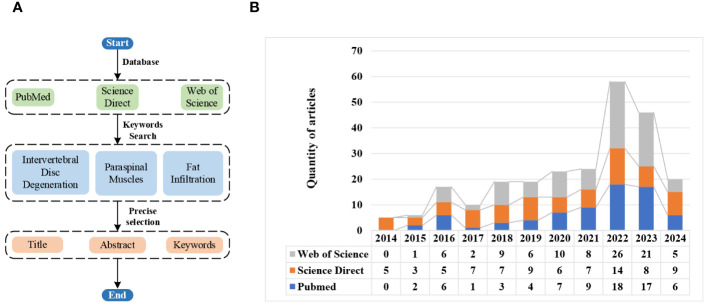
Literature search flowchart and results via different databases. **(A)** Search flowchart. **(B)** Search results.

In recent years, a growing number of studies have reported the interaction between IVDD and fat infiltration of PSMs. To further understand the mechanism of their interaction, relevant literature from 2014 to Apr. 2024 is investigated and counted based on the search keywords of ‘intervertebral disc degeneration, paraspinal muscles, and fatty infiltration’. The specific search process and results are shown in **Figure 1**. From **Figure 1B**, it can be seen that the research on their interaction is generally at a relatively high level, especially in the recent three years.

**Figure 2:**
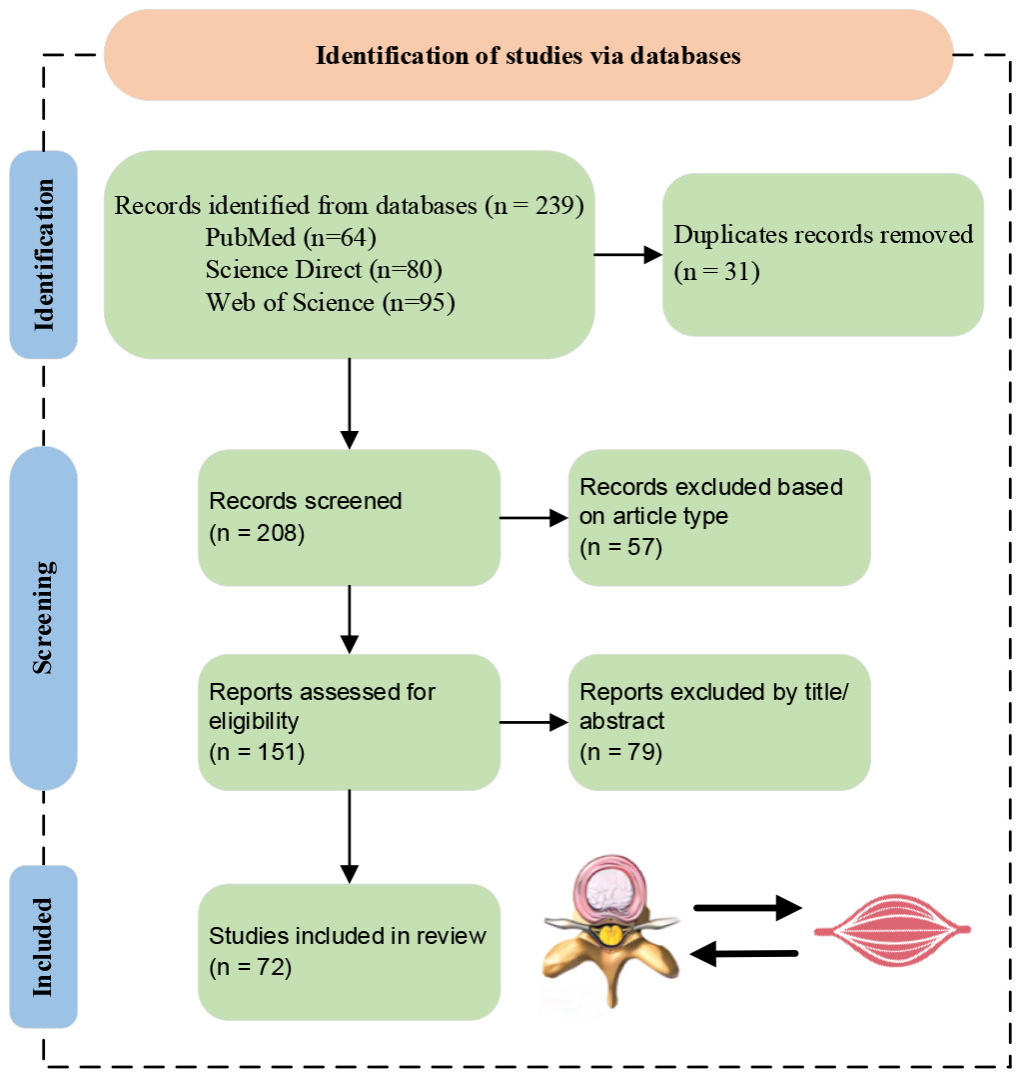
Literature screening flowchart.

Until now, only Wang et al. (24) have published a review on the relationship between IVDD and fat infiltration of PSMs, which has the following shortcomings: (i) Incomplete investigation and analysis on the interaction between IVDD and fat infiltration of PSMs; (ii) Lack of detailed summaries and perspectives. In order to overcome the above shortcomings, this paper offers a comprehensive and in-depth investigation of the interaction between IVDD and fat infiltration of PSMs, aiming to offer a state-of-the-art, one-stop manual for present and further research. Then, the screening process for the review is depicted in **Figure 2**. Inclusion criteria encompassed studies in English focusing on the IVDD and PSMs relationship, with excluded material such as reviews, case reports, and conference abstracts. Finally, a total of 72 papers were included in the investigation. Its main contributions are summarized as follows:

● Four mechanisms of IVDD involved in the fatty infiltration of PSMs and three mechanisms of fatty infiltration of PSMs engaged in IVDD are systematically classified and summarized, upon which the related studies are systematically tabulated and evaluated from four aspects, i.e., methods, conclusions, benefits, and drawbacks.
● In the part of summarizations and discussions, five key findings are put forward from three aspects: research content, methods and results, and their reasons are analyzed.
● Eight perspectives in four main aspects (i.e., promising research, experimental methods, models and samples) are proposed for future research.

The rest of this article is organized as follows: Section 2 summarizes four mechanisms of IVDD involved in fat infiltration of PSMs. Three mechanisms of fat infiltration of PSMs participated in IVDD are concluded in Section 3. Section 4 gives comprehensive summarizations and discussions of some findings. In Section 5, four conclusions are presented. Finally, Section 6 offers several proposals and perspectives for further research.

## 2 Mechanisms of IVDD involved in fat infiltration of PSMs

There are four main mechanisms of IVDD causing fatty infiltration of PSMs, particularly the multifidus muscle (MM): inflammatory response, muscle denervation, muscle chronic disuse, and biomechanical imbalance.

### 2.1 Inflammatory response

Both human and animal studies have consistently demonstrated that dysregulation of local inflammatory activity is a significant contributing factor to the fatty infiltration of PSMs associated with IVDD (12, 15, 19, 20, 25). The summarizations of recent studies exploring this mechanism are presented in **Table 1**. Inflammatory factors play a crucial role in the occurrence and development of IVDD (26, 27). Specifically, tumor necrosis factor-α (TNF-α)and interleukin-1beta (IL-1β), as the most pivotal inflammatory cytokines, exhibit strong pro-inflammatory activity and can stimulate the release of various proinflammatory mediators. Their upregulated expression in degenerative IVD is closely linked to multiple pathological processes of IVDD (28).

**Table 1:** Summary of inflammatory response.

Literature	Year	Methods	Main conclusions	Benefits	Drawbacks
**James et al. (** 25)	2018	Animal experiment	a) During IVDD, macrophages and TNF actively participate in the subacute/early chronic stage of remodeling in muscle, adipose and connective tissues of the MM.	a) Save cost; b) Presents a novel target for treatment.	a) Unknown interference factors may exist in the previous handling of animals; b) Sheep may not be the best animal model for this experiment.
**James et al. (** 20)	2021	Cross-sectional study	a) Increased fatty infiltration of MM is associated with inflammatory dysregulation; b) TNF induces pain and muscle alterations through inflammatory processes in epidural adipose tissue.	a) Provide support for translating observations from animal studies to humans; b) Provide a new mechanism for multifidus muscle changes.	a) Poor sample representativeness; b) Effect of age and BMI on results not excluded; c) Lack of comparison tissue samples; d) Small sample size.
**Shi et al. (** 12)	2022	Retrospective study	a) Fat infiltration of MM may be associated with inflammatory reaction induced by TNF coming from the degenerated IVD; b) Optimal correlation between fatty infiltration of MM and IVDD.	a) Keep consistent baseline demographic characteristics; b) Select patients with NCLBP as control subjects.	a) Select patients with LDH only at L4/L5; b) Small tissue sample size; c) Lack of tissue samples of the pain-free control group.
**Huang et al. (** 15)	2022	Animal experiment and controlled clinical trial	a) Fat infiltration of PSMs and IVDD are causally related; b) Fat infiltration of PSMs is closely related to inflammation, after IVDD.	a) Simple and easy-to-use experimental method; b) Develop a new rat model of DLBP; c) Set up a sham group to exclude puncture interference.	a) Only punctured the L4–6 rat IVD; b) Exist impact on the results from the movement disorder after rat IVD puncture; c) Extraordinary difference between the physiology of humans and rats.
**Chen et al. (** 19)	2023	Prospective cohort study	a) Differences between the ipsilateral and unilateral PSMs were influenced by IVDD and fat infiltration; b) Inflammatory dysfunction is correlated with high-fat filtration and decreased CSA of MM.	a) Provide quantitative data; b) Compare with clinical outcomes.	a) Lack of control group; b) Possible bias and error in tissue sampling; c) Lack of protein expression testing; d) Small sample size.

IVDD, intervertebral disc degeneration; TNF, tumor necrosis factor; MM, multifidus muscle; BMI, body mass index; IVD, intervertebral disc; LDH, lumbar disc herniation; NCLBP, nonspecific low back pain; DLBP, discogenic low back pain; PSMs, paraspinal muscles; CSA, cross sectional area.

IL-1β mediates the production of reactive oxygen species (ROS), and the excessive accumulation of ROS can cause oxidative stress (28). Increased production of ROS is associated with the differentiation of preadipocytes to adipocytes and the accumulation of adipose tissue (29). Persistent oxidative stress can lead to muscle damage, significantly affecting the contractility of skeletal muscle and inducing fibrotic degeneration as well as muscle fatty changes (30). TNF-α is a key player in muscle atrophy, which can induce the upregulation of inflammatory cytokine gene expression and activate the nuclear factor-kappa B (NF-κB) pathway in muscle atrophy (31). Furthermore, TNF-α is related to the disorder of lipid metabolism, and it is potentially an important factor in promoting white adipogenesis in skeletal muscle (22, 28, 32). Interestingly, the relationship between adiposity and pro-inflammatory cytokines is two-way; TNF-α is produced not only by adipose tissue but also regulates adipogenesis (19, 20). In animal models of IVDD or injury, despite no muscle damage, higher expression of proinflammatory cytokine genes (e.g., TNF and IL-1β) and fat infiltration were observed in MM (25, 33–35). The mechanism of fat infiltration in MM is related to the local inflammatory response induced by TNF, which originates from the degenerated disc rather than the PSMs (12). In addition, TNF expression in MM after IVDD is mediated by M1 macrophages in adipose tissue (25).

Fibroblasts, preadipocytes, and satellite cells are existent in the perimuscular connective tissue. These cells can transdifferentiate into adipose tissue under the stimulation of pro-inflammatory cytokines, ultimately leading to adipocyte proliferation (30). A large number of fibro-adipogenic progenitors (FAPs) and satellite cells (SCs) were found in the MM of patients with lumbar disc herniation (LDH) (36). FAPs are an inherent muscle stem cell population that differentiate into adipocytes and fibroblasts and contribute significantly to muscle fat infiltration and fibrosis (37, 38). FAPs appear as a brown/beige fat (BAT) phenotype, whereas white adipose tissue makes up the majority of muscle fat infiltration (39, 40). BAT has demonstrated positive effects on myogenesis (41). Therefore, a potential avenue for mitigating fat infiltration (FI) and muscle atrophy in the future involves directing FAPs toward a BAT phenotype.

**Figure 3:**
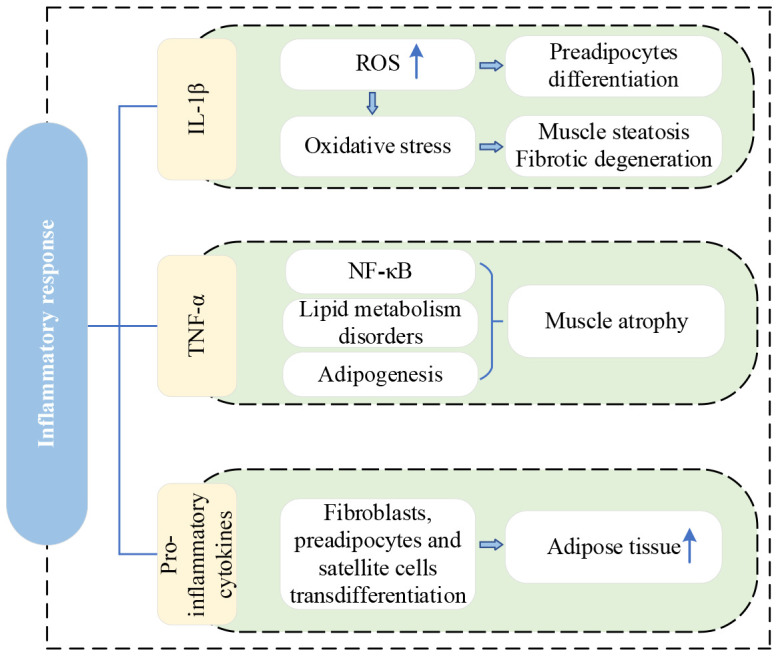
Research lineage of inflammatory response. IL-1β, interleukin-1beta; ROS, reactive oxygen species; TNF-α, tumor necrosis factor-α; NF-κB, nuclear factor-kappa B.

**Figure 3** shows the current lineage of research on inflammatory response.

### 2.2 Muscle denervation

IVDD is a complex process involving molecular and structural alterations, which causes the compression of both the dorsal root ganglia and the nerve root (42), with atrophy and fat infiltration in the PSMs they innervate (17, 18, 43, 44). Recent studies on this mechanism are summarized in **Table 2**. Muscle denervation usually undergoes three stages: 1) immediate loss of function, followed by rapid weight loss and muscle fiber atrophy, 2) more severe muscle atrophy, including loss of most sarcomeric tissue, and 3) interstitial fibrosis and fat deposition (45). Additionally, denervated muscles experience alterations in glucose metabolism and protein hydrolysis, which are associated with reduced muscle fiber diameter and increased fat content. A review of the MRI appearance of muscle denervation by Kamath et al. reveals that progressive muscle atrophy and fat infiltration can be observed a few weeks after denervation (46). Consequently, in the absence of neural innervation, skeletal muscles inevitably undergo a process of atrophy, eventually largely replaced by fibrous connective and adipose tissue. Similarly, continuous compression of a nerve root by disc herniation also leads to muscle atrophy and fat replacement in muscles supplied by that nerve (47, 48). The MM is particularly susceptible because of being innervated only by the medial branch of the dorsal root of the spinal nerve, with no intersegmental nerve supply like other PSMs (44). Early studies demonstrated that denervated rabbit muscles can transform into white adipose tissue and express specific genes, especially the adipose obese (ob) gene (49). The expression of the ob gene is specific to adipose tissue in various animal species, including humans. Moreover, denervated skeletal muscles of most animals exhibit a dramatic increase in DNA synthesis, since the proliferation of all kinds of cells such as satellite cells, macrophages, mast cells, etc. Most of these cells secrete various cytokines to stimulate the proliferation and differentiation of macrophages, mast cells, fibroblasts, preadipocytes, and muscle precursor cells, thereby promoting muscle repair or fat degeneration (49). Additionally, it has been shown that TNF-α influences myoblast differentiation and fiber degradation and its expression increases with disc injury and affects axon conduction (50).

**Table 2:** Summary of muscle denervation.

Literature	Year	Methods	Main conclusions	Benefits	Drawbacks
**Hodges et al. (** 17)	2006	Animal experiment	a) The cross-sectional area of MM decreases and adipocytes enlarge after disc and nerve lesions; b) Changes after disc lesion affect one level, which may be due to disuse following reflex inhibitory mechanisms.	a) Set up a sham group to exclude operative procedure interference; b) A new reliable method of measuring muscle cross-sectional area.	a) The anterolateral annular lesion is not the best model to study the effects of disc injury; b) Differences in muscle response and mechanisms to injury between pigs and humans.
**Battie et al. (** 43)	2012	Cross-sectional study	a) Within 6 weeks of the appearance of radicular symptoms related to disc herniation, the MM shows fatty infiltration rather than CSA asymmetry.	a) Quantitative measurements of muscle CSA and signal using MRI; b) Minimize the impact of confounding factors.	a) Cannot exclude the possibility that muscle asymmetry existed before the onset of symptoms; b) Lack of evidence to confirm nerve root involvement and denervation; c) Possible measurement bias.
**Yaltirik et al. (** 44)	2018	Case-control study	a) Long-term pressure of nerve roots by disc herniation can cause muscle atrophy and degeneration; b) Muscle degeneration can be detected by decreased cross-sectional area and increased fat deposition.	a) Considered gender and age; b) Independent observer measurement.	a) Not exclude the effect of potential factors such as body weight on the cross-sectional area of PSMs.
**Boström et al. (** 18)	2022	retrospective study	a) Increased fat infiltration in MM and longissimus muscle in dogs with compressive IVDD relative to non-compressive nucleus pulposus extrusion, likely resulting from denervation and secondary disuse caused by spinal cord compression and pain.	a) Reliable measurement method; b) Measurement of several slices from the same segments.	a) Impossible to compare with healthy dogs; b) May have clinical signs in IVDD compression dogs before the scan; c) Lack of information on exercise level and parameters; d) Influence of transverse slice in cross-sectional area measurement.

MM, multifidus muscle; CSA, cross sectional area; PSMs, paraspinal muscles; IVDD, intervertebral disc degeneration.

**Figure 4:**
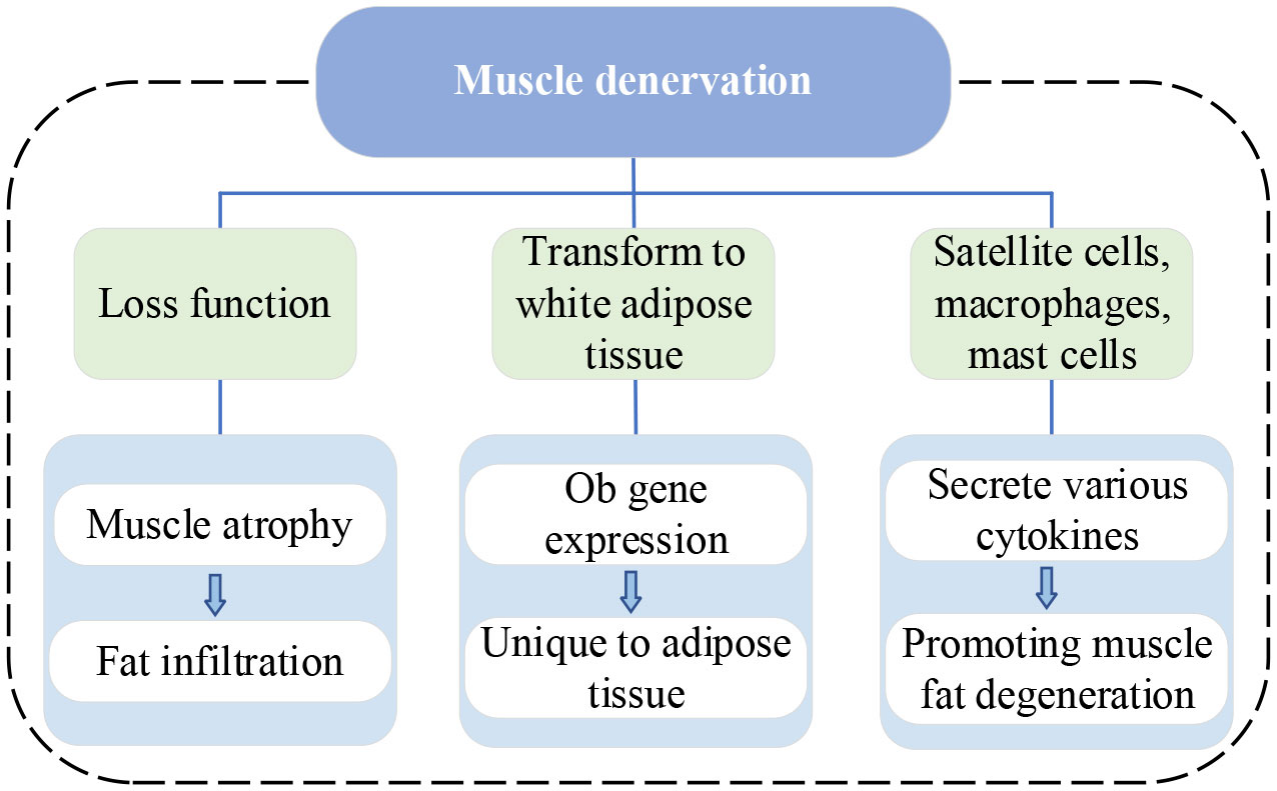
Research vein of muscle denervation. ob, adipose obese.

**Figure 4** illustrates the current vein of research on muscle denervation.

### 2.3 Muscle disuse

IVDD is the main cause of LBP and significantly affects the stability and motor function of the spine. Pain and discomfort from IVDD often make individuals reduce exercise and activity, causing the disuse of PSMs – a lack of exercise. Short-term disuse promotes fatty acid infiltration into skeletal muscle cells and is associated with the development of intermuscular adipose tissue(IMAT) (51). **Table 3** provides a summary of recent studies on this mechanism. When PSMs lack exercise, their capillary reactivity decreases, resulting in the muscle blood supply being inadequate and leaving the muscle in a hypoxic state (56). Persistent hypoxia causes incomplete utilization of muscle glycogen and accumulation of lactic acid and various metabolites, which induces muscle edema, stimulating or exacerbating nerve pain (57, 58). The long-term effect makes the muscle gradually thin and degenerate, eventually replaced by adipose tissue (59). Previous studies have indicated a higher accumulation of adipose tissue in PSMs is related to physical inactivity (54, 60). Movement and gravitational loading are essential for maintaining a healthy spine. When the axial load of the human body decreases, such as during prolonged bedrest or spaceflight, the active and passive elements of the lumbar spine are affected (61). In response to decreased use, the lumbar spine undergoes adaptive remodeling (62). Adverse effects include lumbopelvic muscle atrophy, fat accumulation in PSMs, reduced lumbar lordosis, and altered disc hydration status (54, 61). Surprisingly, Hides et al. (63) observed a significant increase in the CSA of the MM during the last magnetic resonance scan of bed rest and the first scan of re-loading. They suggested that this alteration might be attributed to fluid transfer of the atrophic muscle during reloading or fiber and connective tissue damage (subsequent swelling) after walking to mask muscle atrophy. The double S-curve of the spine enables the body to slow down the vertical gravitational loads and spread forces horizontally. However, this requires the action of muscles in the lumbopelvic region to maintain the anterior kyphosis of the spine, especially the MM (64). Bed rest eliminates axial gravitational loading from the spine, potentially depriving the necessary stimulus for the normal functioning of the MM (63). In addition, disc degeneration itself can cause the physiological curve of the spine to become shallower and disappear.

**Table 3:** Summary of muscle disuse.

Literature	Year	Methods	Main conclusions	Benefits	Drawbacks
**James et al. (** 52)	2018	Longitudinal case-control animal model	a) The sub-acute/early chronic phase of remodeling in the multifidus and muscle hyperalgesia are driven by inflammatory processes; b) The inflammatory response (i.e. expression of IL-1β and adiponectin) in muscle secondary to IVDD was prevented by exercise.	a) Provide four new insights about IVDD in regulating inflammatory pathways in MM; b) Offer a new chance to study the inflammatory profile of MM near spontaneous IVDD.	a) Lack the time course information from the onset of IVDD; b) Differences in the pathological mechanisms between humans and rats.
**Maurer et al. (** 53)	2020	Cross-sectional case-control study	a) The degree of physical inactivity observed over 14 14-year period showed a strong correlation with IVDD of the thoracic and lumbar spine.	a) Large sample size; b) A 14-year observation period.	a) Limited MRI dates with no earlier images for comparisons; b) Exclusion of chronic back pain patients; c) Only correlation without a causal relationship was investigated; d) Lack of physical activity details.
**De Martino et al. (** 54)	2022	Prospective longitudinal study	a) Accumulation of ILC and inhomogeneous spatial distribution were found in the lumbar musculature after 60 days of bed rest; b) Provide a new target for lumbar muscle repair for those exposed to prolonged extreme physical inactivity, e.g., astronauts, the elderly, or individuals with chronic LBP.	a) Quantify the spatial pattern of ILC accumulation.	a) Small sample size; b) Complex and costly research; c) Limitations of muscle segmentation methods.
**Wesselink et al. (** 55)	2023	Review article	a) Moderate-quality evidence suggests that paraspinal fat infiltration cannot be reversed by exercise in patients with LBP; b) To draw definite conclusions, more studies with consistent, and consensus-driven, methodologies, larger sample sizes and longer treatment duration are required.	a) Compared with early reviews; b) Constructive perspectives for future tasks.	a) A small number of available studies; b) Limited to the pool and compare data from different studies; c) Provide moderate-quality evidence.

IL-1β, interleukin-1beta; IVDD, intervertebral disc degeneration; MRI, magnetic resonance imaging; ILC, intramuscular lipid concentration.

Brisk walking (65), aerobic training (66), resistance training (67–69), and other forms of exercise can effectively prevent fat infiltration or delay its progression speed. Additionally, exercise not only prevents but also improves the inflammatory response in muscles secondary to IVDD (52). This positive effect is attributed to exercise promoting the release of immunomodulatory factors in the blood, which exerts anti-inflammatory effects. Interestingly, Wesselink et al.’s review shows that fat infiltration of PSMs in patients with LBP is irreversible through exercise (55). However, it’s important to note that their conclusions are based on studies with low statistical power and methodological quality, and only provide moderate-quality evidence. In the future, more and higher quality research is needed to draw definitive conclusions.

**Figure 5:**
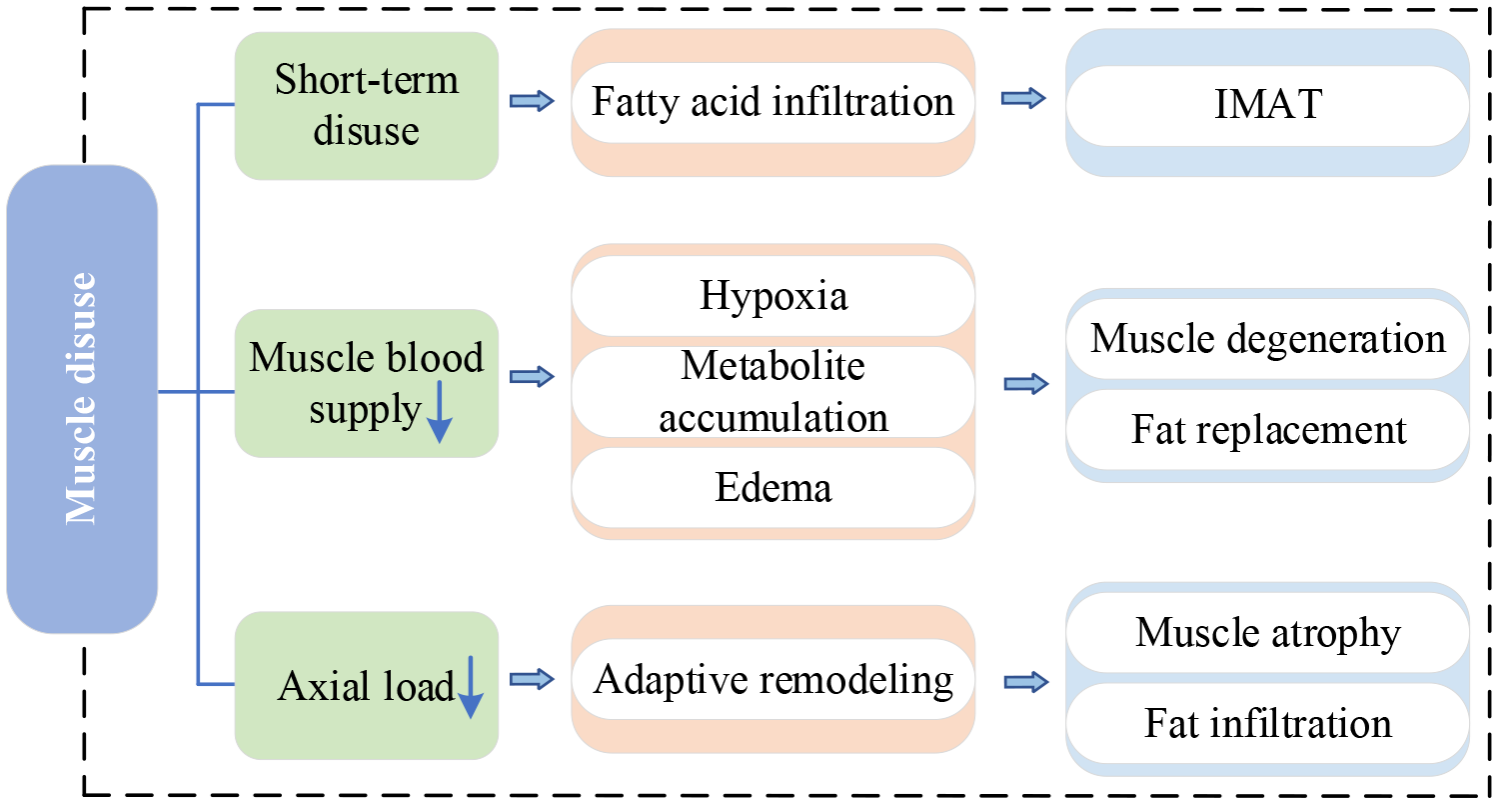
Research network of muscle disuse. IMAT, intermuscular adipose tissue.

**Figure 5** provides the current research network on muscle disuse.

### 2.4 Biomechanical imbalance

The IVD and PSMs are not only surface adjacent but also biomechanical and biochemical interconnected (70). Serving as a crucial structure in the spine, the IVD offers buffering and support between adjacent vertebrae but is susceptible to degeneration influenced by factors such as age, lifestyle, and genetics. After degeneration, IVD loses its normal structure and function, which leads to the narrowing of the disc space and settling of the motion segment (71). Subsequently, the ligamentum flavum buckles, eventually leading to instability of the lumbar spine. The main function of PSMs is to maintain the stability and movement of the spine. Under normal circumstances, the load on PSMs in humans is in balance. However, when IVDD causes changes in disc height and vertebral instability, the tension load on PSMs increases to maintain spinal stability. If instability persists or becomes severe, surpassing the stress limits of the PSMs, it can result in atrophy and fat infiltration (16, 71, 72).

**Figure 6:**
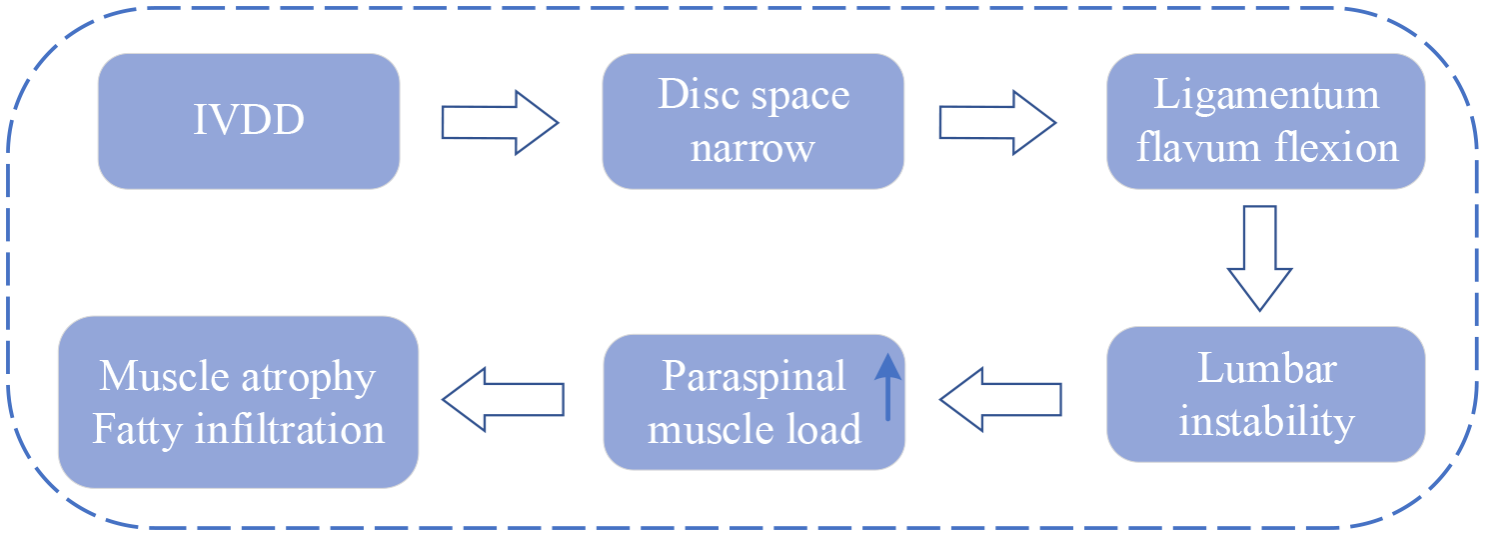
Research context of biomechanical imbalance. IVDD, intervertebral disc degeneration.

The current context of research on biomechanical imbalances is shown in **Figure 6**.

## 3 Mechanisms of fat infiltration of PSMs involved in IVDD

Similar to the impact of IVDD on PSMs, fat-infiltrated PSMs also affect IVD. However, there are few studies on the impact of fat infiltration of PSMs in the IVDD. Here we summarize three mechanisms, inflammation, lipid metabolism disorders, and abnormal mechanical load.

### 3.1 Inflammation mechanism

Adipose tissue is a primary source of numerous inflammatory cytokines, and an excess of adipocytes contributes to a state of systemic chronic low-grade inflammation. Previous studies have established a link between proinflammatory cytokines derived from adipose tissue(including TNF and IL-Iβ) and both obesity and osteoarthritis (73, 74). Obesity can cause fatty infiltration of PSMs (75). Furthermore, in obesity, paraspinal adipose tissue is considered to be a source of systemic pro-inflammatory cytokines (19). The fat infiltration of PSMs primarily consists of white adipose tissue, and the abnormal accumulation of fat leads to functional impairment of white adipose tissue, characterized by elevated levels of specific pro-inflammatory cytokines such as TNF (22). In addition, adipocytes in white adipose tissue recruit macrophages from the blood by synthesizing and secreting lactic acid, and the macrophages are polarized after infiltrating into fat, forming CD11c+ M1 type, highly expressing pro-inflammatory cytokines such as IL-1β and TNF (76), which are key driving factors of IVDD and closely associated with various pathological processes of IVDD (28).

TNF-α primarily exerts its effects through activating the NF-κB signaling pathway, which causes the expression of type II collagen and proteoglycan to be downregulated, and the expression of a disintegrin and metalloproteinase with thrombospondin motifs (ADAMTs)-4,5, matrix metalloproteinases (MMPs) to be upregulated. This cascade induces matrix degradation, cellular apoptosis, and precipitating degenerative changes in the lumbar vertebral discs (77). Simultaneously, the death domain of TNF receptors binds to other intracellular signaling proteins containing death domains, such as TNF receptor-associated death domain protein (TRADD) and Fas-associated death domain protein (FADD). This interaction triggers the cysteinyl-aspartate-specific protease (caspase) family of cascade reactions, which leads to caspase-3 activation, ultimately causing cell apoptosis (78). Additionally, TNF-α also directly inhibits NP cell activity and reduces the expression of growth differentiation factor 5(GDF-5) to accelerate IVDD.

IL-1β mainly activates mitogen-activated protein kinase(MAPK) and nuclear factor-kappa B(NF-κB) signaling pathways, upregulating MMPs-1, 2, 3, 4, 13, and ADAMTs-4, 5 (79). This leads to the degradation of type II collagen and proteoglycans, thereby accelerating the development of IVDD. Furthermore, IL-1β stimulates the expression of vascular endothelial growth factor(VEGF) and nerve growth factor(NGF), promoting the invasion of nerves and blood vessels into degenerated discs (80), eventually inducing LBP. Beyond these effects, IL-1β amplifies the inflammatory cascade by increasing the secretion of inflammatory cytokines (IL-6, 8, 17) and C-C Motif Chemokine Ligand chemokines (CCL-2, 3, 5, 7). This, in turn, attracts immune cells to accumulate at the site of inflammation, releasing inflammatory mediators that exacerbate IVDD (81–83).

**Figure 7:**
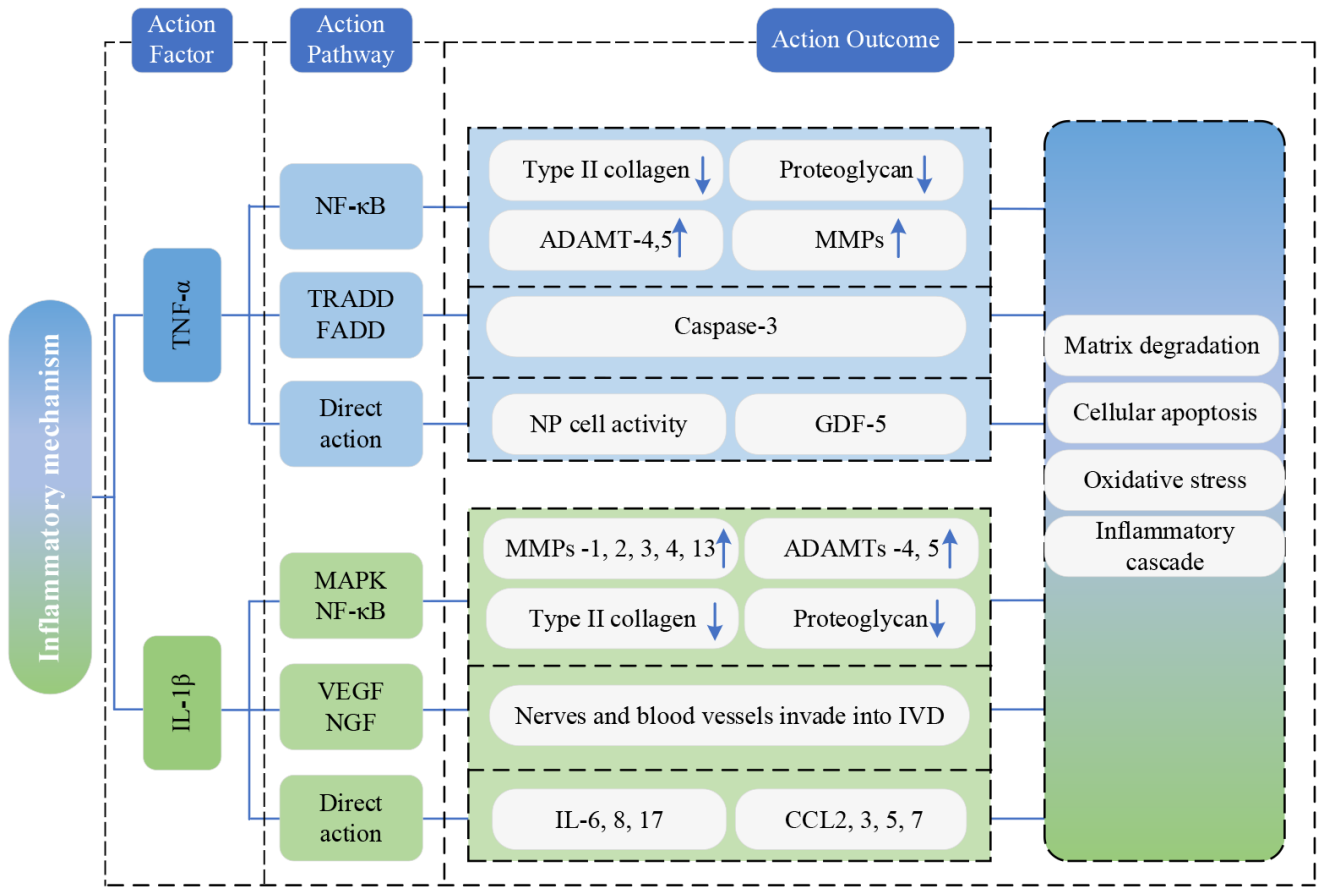
Research state of Inflammation mechanism. TNF-α, tumor necrosis factor-α; NF-κB, nuclear factor-kappa B; IL-1β, interleukin-1beta; ADAMTs, a disintegrin and metalloproteinase with thrombospondin motifs; MMP, matrix metalloproteinases; TRADD, TNF receptor-associated death domain protein; FADD, Fas-associated death domain protein; NP, nucleus pulposus; GDF-5, growth differentiation factor 5; MAPK, mitogen-activated protein kinase; NF-κB, nuclear factor-kappa B; MMPs, matrix metalloproteinases; ADAMTs, a disintegrin and metalloproteinase with thrombospondin motifs; VEGF, vascular endothelial growth factor; NGF, nerve growth factor; IVD, Intervertebral disc; IL, interleukin; CCL, C-C Motif Chemokine Ligand.

**Figure 7** portrays the present state of research on the inflammation mechanism.

### 3.2 Lipid metabolism disorder

Adipocyte-secreted adipokines, as biologically active substances with metabolic and immunomodulatory functions, play a significant role in the occurrence and progression of IVDD (23).

Leptin can stimulate the proliferation of AF and NP cells by inducing the expression of cell cycle protein D1 and activating JAK-STAT3, MEK-ERK and PI3K-Akt signaling pathways (84). However, despite this proliferative effect, the increased expression of extracellular matrix-degrading enzymes prevents the proper synthesis of extracellular matrix components by the proliferating cells. This imbalance promotes catabolism and contributes to disc cell senescence, eventually leading to IVDD (84, 85). Additionally, when leptin acts alone or in synergy with TNF-α, IL-1β, or IL-6 in NP cells, it significantly increases the production of NO and expression of inflammatory cytokines and MMPs, speeding up the process of IVDD (86). Leptin can also promote calcification of the CEP, interfering with the transport of nutrients to disc cells, and ultimately contributing to IVDD (87).

Adiponectin levels are related to IVDD, nevertheless, conflicting results have been reported in published literature. Khabour et al. discovered higher levels of circulating adiponectin in IVDD patients compared to healthy controls (88), while Yuan et al. verified a downregulation of adiponectin expression in IVDD patients, which is negatively correlated with IVDD severity (89). In addition, adiponectin performs anti-inflammatory effects by inhibiting the expression of pro-inflammatory mediators (89). Therefore, the downregulation of adiponectin in IVD may lead to an imbalanced inflammatory response and contribute to disc degeneration (84).

Resistin is upregulated in IVDD and positively correlates with the Pfirrmann grade (90). Through activating p38-MAPK and NF-κB signaling pathways, resistin could bind to Toll-like receptor 4(TLR4) and augment the expression of CCL4 in NP cells, promoting macrophage infiltration and inflammatory response in IVD tissue (90). Furthermore, resistin, by activating the p38-MAPK signaling pathway, induces metabolic disruption in NP cells and accelerates the progression of IVDD (91).

Visfatin is associated with cell differentiation, stress, apoptosis, and inflammation processes (92). In NP cells, IL-1β stimulates the expression of visfatin, increasing degradation-related proteins ADAMTs-4,5 and MMPs-3,13 and decreasing type II collagen and aggrecan. This process prompts the degradation of the extracellular matrix (22). Moreover, the expression level of visfatin is positively correlated with the degree of IVDD (93).

**Figure 8:**
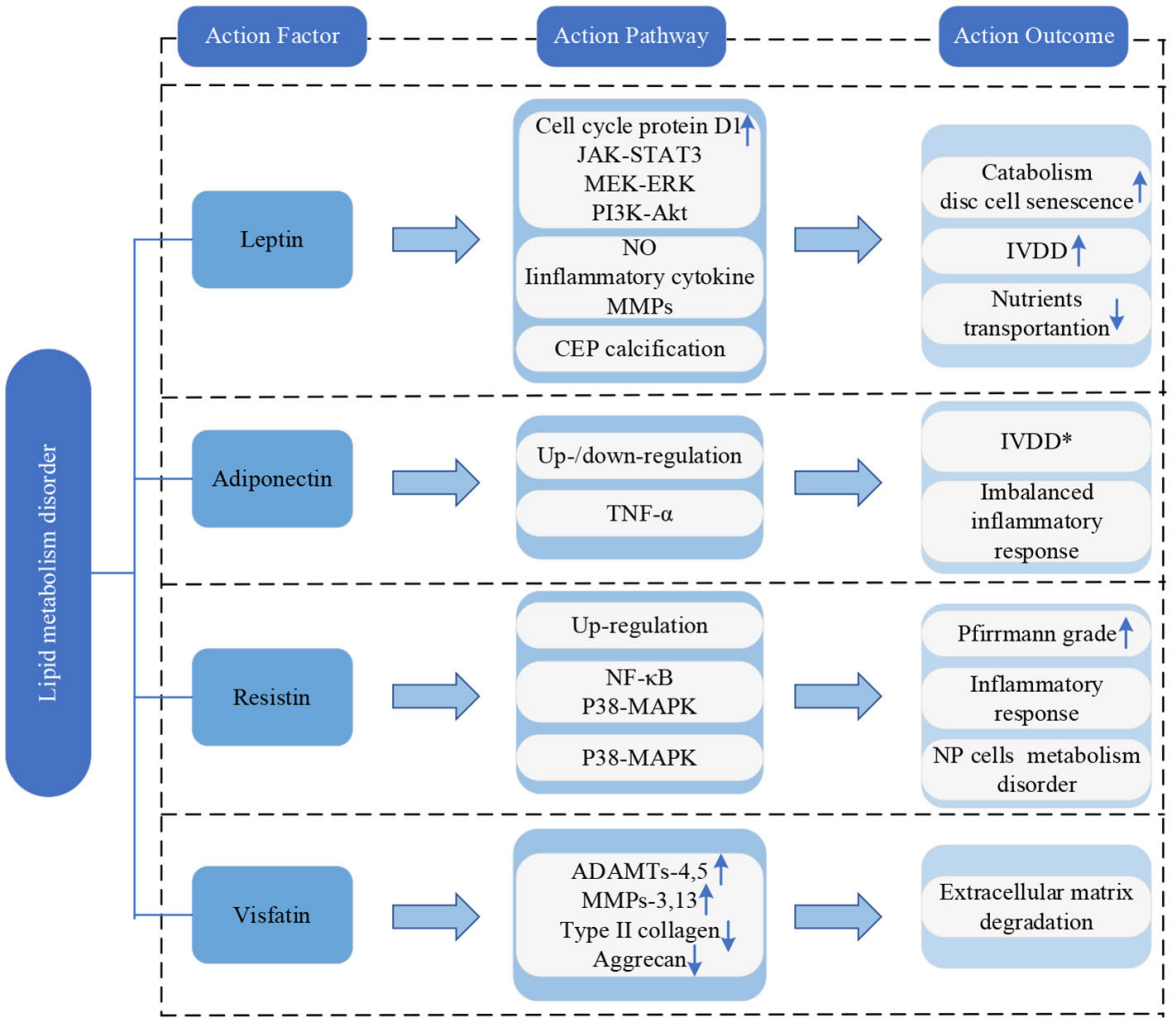
Research framework of lipid metabolism disorder. IVDD, intervertebral disc degeneration; CEP, cartilaginous endplate; TNF-α, tumor necrosis factor-α; NP, nucleus pulposus; NF-κB, nuclear factor-kappa B; MAPK, mitogen-activated protein kinase; ADAMTs, a disintegrin and metalloproteinase with thrombospondin motifs; MMPs, matrix metalloproteinases. * indicates controversy.

The current framework of research on adipokines is depicted in **Figure 8**.

### 3.3 Abnormal mechanical stress

Spinal instability is the premise of the main pathological mechanism leading to IVDD (94). PSMs are a major source of load-bearing for the spine and play a crucial role in its movement and stability. When the PSMs are replaced by fatty tissue, the contractility and load-bearing capacity of muscles are weakened. Consequently, more pressure is transferred to the discs and small joints (21). If the pressure load is excessively high or exits for too long, NP cells will age, apoptosis, and necrosis, resulting in lower activity and decreased numbers, which is one of the most important pathological processes in IVDD (95). Abnormal pressure load can also accelerate the calcification and vascular degeneration of the CEP, leading to a significant decrease in permeability coefficients and transport rates (42). This makes it more difficult for sugar, oxygen, and other macromolecular nutrients IVD cells require and metabolites such as lactic acid to diffuse or excrete. In the acidic and hypoxic microenvironment, the activity and numbers of NP cells decreased, with weakened extracellular matrix (ECM) anabolism capacity, as a result promoting the progress of IVDD (96, 97). Additionally, abnormal mechanical loads can induce increased expression of MMPs and ADAMTs genes, so that the catabolism of ECM exceeds the anabolism. This results in the content of type II collagen and proteoglycan decreasing significantly, leading to dehydration and fibrosis of NP tissue, a reduction in intervertebral space height, instability of the IVD structure, and an accelerated progression of IVDD (98).

**Figure 9:**
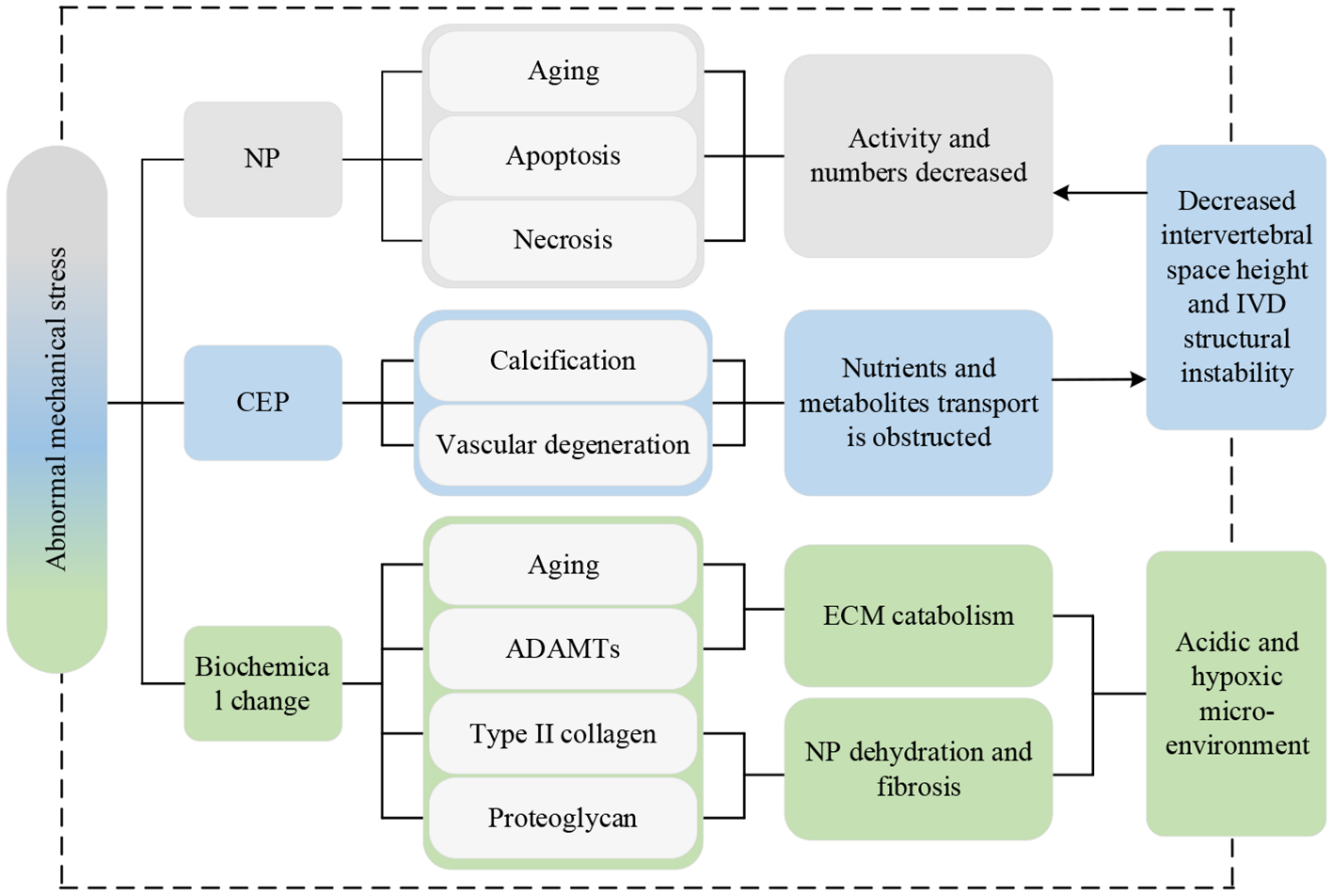
Research lineage of abnormal mechanical stress. Np, nucleus pulposus; CEP, cartilaginous endplate; IVD, intervertebral disc; ADAMTs, a disintegrin and metalloproteinase with thrombospondin motifs; ECM, extracellular matrix.

The current lineage of research on abnormal mechanical stress is depicted in **Figure 9**.

## 4 Summary of the evidence and discussion

**Figure 10:**
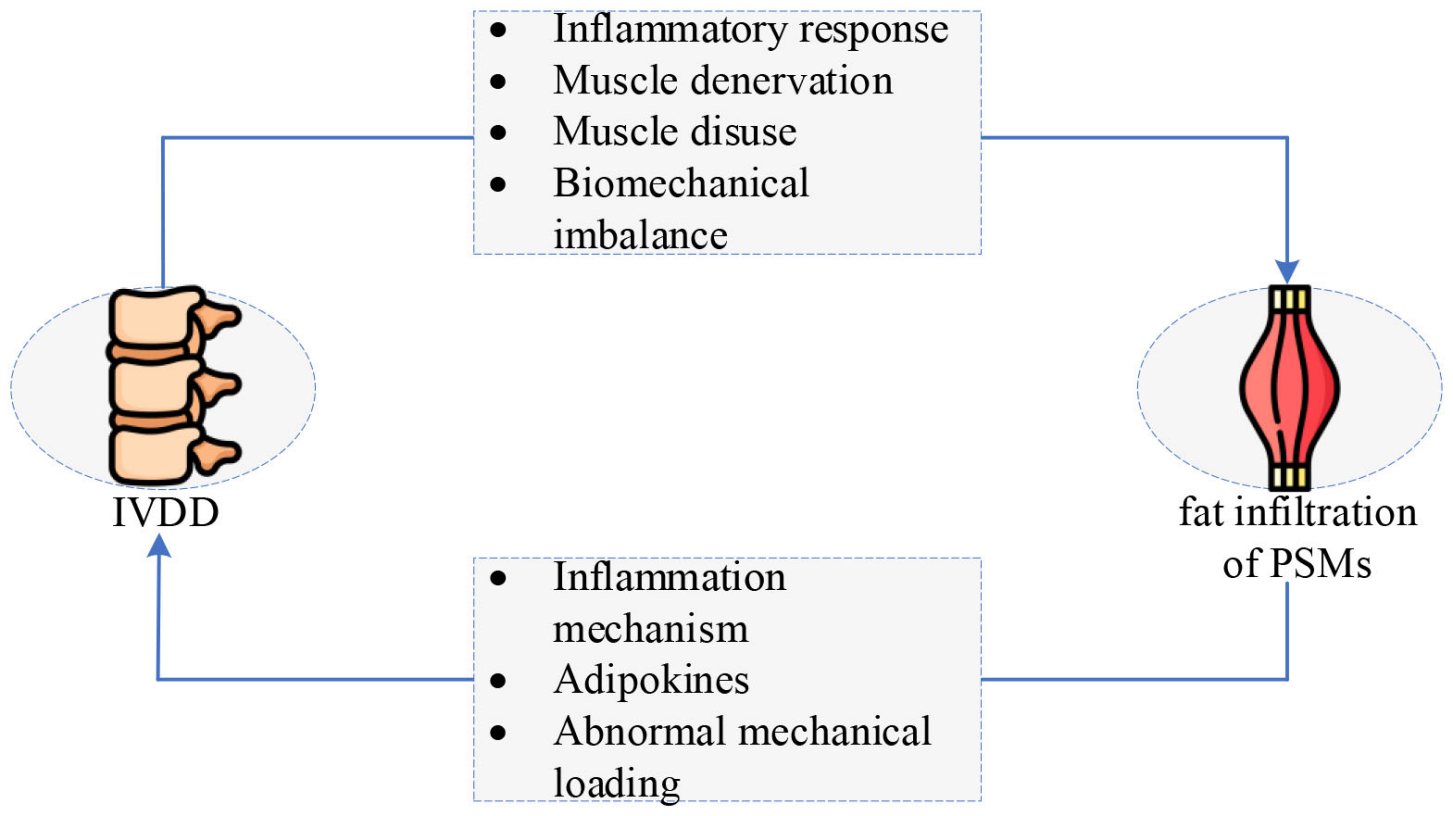
The interaction between IVDD and fat infiltration of PSMs: a vicious circle. IVDD, intervertebral disc degeneration. (The original image is from https://www.freepik.com/pricing.).

**Figure 10** depicts the above mechanisms intuitively, illustrating more distinctly and visually the interaction between IVDD and fat infiltration of PSMs. Here, several summaries and discussions are stated as follows:

a) Among all the above mechanisms, inflammation is the most extensively studied, because of the universality of the inflammatory response and the numerous types and functions of inflammatory factors.
b) Most studies concentrate on fatty infiltration of the MM. The reason is that MM has the largest attachment area and is only innervated by a single nerve, making it more susceptible and simple to study (99–101).
c) The degree and incidence of IVDD are higher at the L4-L5 and L5-S1 levels, since the proximity of the distal lumbar segments to the static sacrum, and their mobility and stresses are greater than the sacral segments, which is conducive to degeneration (102).
d) The research methods mainly focus on animal experiments and cross-sectional studies, it may be a choice that strategically considers factors such as time efficiency, cost-effectiveness, and the feasibility of repetition.

## 5 Conclusions

This paper systematically reviews the interaction between IVDD and fat infiltration of PSMs and classifies its mechanisms. Here, four main conclusions are drawn as follows:

a) IVDD and fat infiltration of PSMs can promote the occurrence and development of each other, forming a vicious cycle that ultimately leads to LBP and disability.
b) Four mechanisms of IVDD causing fatty infiltration of PSMs are summarized, i.e., inflammatory response, muscle denervation, muscle disuse, and biomechanical imbalance.
c) Three mechanisms of IVDD caused by fatty infiltration in PSMs are systematically reviewed: inflammation mechanism, lipid metabolism disorder, and abnormal mechanical stress.
d) Inflammation functions as a bridge between IVDD and fat infiltration of PSMs. Exercise can prevent and improve the inflammatory response.

## 6 Perspectives

Eight proposals and perspectives in four main aspects (i.e., promising research, experimental methods, models and samples) are proposed as follows:

a) The molecular-level interaction between intervertebral disc tissue and paraspinal muscle fat is not yet fully understood. Specifically, the molecular mechanisms of signal transduction and cytokine release between intervertebral disc cells and paraspinal muscle adipocytes still need to be further studied.
b) Muscles at a single spinal level do not represent the whole lumbar spine muscles, so future studies should measure the amount of fat infiltration in each spine-level muscle and collect its tissue samples.
c) Differences in the segmentation method of fat infiltration in PSMs (in- or excluding epimuscular fat) in different studies, thus clear quantification of lipid levels within and out of muscle cells is recommended for future research, ensuring more consistent and comparable outcomes in the studies assessing fat infiltration of PSMs.
d) Although rats are a commonly used experimental animal model, they cannot fully simulate humans due to the huge differences between them and humans. Hence, animal models closer to humans should be explored, or high-quality clinical cohort studies could be conducted.
e) Given the complexity, time, and cost of research, numerous studies, both animal experiments and clinical investigations often have relatively small sample sizes. Therefore, future studies could increase the sample size to obtain more powerful and reliable results.
f) Considering the pivotal role of PSMs’ health in spinal stability and functional outcomes, it is recommended to integrate information concerning the extent of fat infiltration and atrophy of PSMs into decision-making algorithms for Early Recovery After Surgery (ERAS) protocols. According to a systematic review by Zaed et al. (103), the importance of ERAS in spinal surgery is emphasized but still needs to be supported by high-quality clinical randomized controlled trials (RCT). Therefore, it is also advised that fat infiltration be taken into account in future RCT.
g) The treatment of LBP should pay more attention to the relationship between IVD and PSMs. Given the complex interplay between IVDD and fat infiltration of PSMs, a multimodal treatment targeting multiple mechanisms can be adopted in the future. Combining pharmacotherapy, exercise interventions, and neuromodulation techniques may offer synergistic benefits in managing LBP.
h) Considering that inflammation is a critical bridge between IVDD and fatty infiltration of PSMs, it may be possible in the future to disrupt the vicious cycle of IVDD and fatty infiltration and halt the progression of LBP with targeted anti-inflammatory drug therapy.

## Author contributions

JJ: Writing – original draft, Writing – review & editing, Formal analysis, Investigation, Visualization. YH: Funding acquisition, Conceptualization, Writing – review & editing. BH: Funding acquisition, Supervision, Validation, Writing – review & editing.
